# Accuracy of Continuous Glucose Monitoring (CGM) during Continuous and High-Intensity Interval Exercise in Patients with Type 1 Diabetes Mellitus

**DOI:** 10.3390/nu8080489

**Published:** 2016-08-10

**Authors:** Othmar Moser, Julia K. Mader, Gerhard Tschakert, Alexander Mueller, Werner Groeschl, Thomas R. Pieber, Gerd Koehler, Janin Messerschmidt, Peter Hofmann

**Affiliations:** 1 Institute of Sports Sciences, Exercise Physiology, Training & Training Therapy Research Group, University of Graz, Max-Mell-Allee 11/III, Graz 8010, Austria; othmar.moser@hotmail.com (O.M.); gerhard.tschakert@uni-graz.at (G.T.); alexander.mueller@uni-graz.at (A.M.); werner.groeschl@gmail.com (W.G.); 2 Department of Internal Medicine, Division of Endocrinology & Diabetology, Medical University of Graz, Auenbruggerplatz 15, Graz 8036, Austria; julia.mader@medunigraz.at (J.K.M.); thomas.pieber@medunigraz.at (T.R.P.); gerd.koehler@klinikum-graz.at (G.K.); 3 Institute of Health and Tourism Management, Sports Science Laboratory, FH JOANNEUM—University of Applied Sciences, Kaiser-Franz-Josef Straße 24, Bad Gleichenberg 8344, Austria; 4 Center of Sports Medicine & Sports Orthopedics, University Outpatient Clinic, University of Potsdam, Am Neuen Palais 12, Potsdam 14469, Germany; 5 Department of Haematology, Oncology and Palliative Care, Klinikum Ernst von Bergmann, Charlottenstraße 72, Potsdam 14467, Germany; janin.messerschmidt@gmx.de

**Keywords:** continuous glucose monitoring, exercise, diabetes, blood glucose

## Abstract

Continuous exercise (CON) and high-intensity interval exercise (HIIE) can be safely performed with type 1 diabetes mellitus (T1DM). Additionally, continuous glucose monitoring (CGM) systems may serve as a tool to reduce the risk of exercise-induced hypoglycemia. It is unclear if CGM is accurate during CON and HIIE at different mean workloads. Seven T1DM patients performed CON and HIIE at 5% below (L) and above (M) the first lactate turn point (LTP_1_), and 5% below the second lactate turn point (LTP_2_) (H) on a cycle ergometer. Glucose was measured via CGM and in capillary blood (BG). Differences were found in comparison of CGM vs. BG in three out of the six tests (*p* < 0.05). In CON, bias and levels of agreement for L, M, and H were found at: 0.85 (−3.44, 5.15) mmol·L^−1^, −0.45 (−3.95, 3.05) mmol·L^−1^, −0.31 (−8.83, 8.20) mmol·L^−1^ and at 1.17 (−2.06, 4.40) mmol·L^−1^, 0.11 (−5.79, 6.01) mmol·L^−1^, 1.48 (−2.60, 5.57) mmol·L^−1^ in HIIE for the same intensities. Clinically-acceptable results (except for CON H) were found. CGM estimated BG to be clinically acceptable, except for CON H. Additionally, using CGM may increase avoidance of exercise-induced hypoglycemia, but usual BG control should be performed during intense exercise.

## 1. Introduction

Physical activity and exercise training have become greater focuses of interest as an additional therapy tool in the treatment of diabetes [1,2]. A physically active lifestyle is generally associated with decreases in all-cause mortality and cardiovascular disease risk [3]. Although, exercise in type 1 diabetes mellitus (T1DM) is also linked to a drastic drop of blood glucose (BG) during exercise, which increases the risk of hypoglycemia [4].

Continuous glucose monitoring (CGM) systems were mostly used to analyze pre- and post-exercise glucose levels to achieve a deeper understanding of the long-term effects of exercise on glucose response [5,6]. Additionally, CGM is also used to reduce the risk of exercise-induced hypoglycemia. Even under different glycemic conditions (euglycemia, hypoglycemia, and hyperglycemia), that might be similar to those during exercise, CGM is able to accurately track acute changes in glucose concentration [7]. Few studies investigated the accuracy of CGM systems during exercise in healthy [8] or T1DM subjects [9,10,11,12,13]. In a study by Yardley et al. [9] patients with T1DM, displayed an acceptable comparison of a CGM system with plasma glucose during aerobic exercise. Studies on the effects of non-continuous exercise workloads are rare and have methodological limitations [11,13]. The mean exercise intensity (P_mean_) of continuous (CON) and high-intensity interval exercise (HIIE) was not matched [11] or only HIIE was performed [13]. In the study of Bally et al. [11] the accuracy of a CGM system was investigated in ten patients with T1DM during cycling at 50% of peak oxygen uptake, either with or without a 10 s all-out sprint every 10 min. Although they found comparable accuracy of CGM system during CON and HIIE, different metabolic conditions (lactate 7.3 ± 0.5 vs. 2.6 ± 0.3 mmol·L^−1^) were observed. To make any conclusion regarding the influence of each type of exercise, it is necessary to match P_mean_ of CON and HIIE. Without matching P_mean_ two variables, such as type of exercise and mean load, can influence the outcome, making it unlikely to differentiate the true cause of the effect. In a pilot study of Iscoe et al. [13] a spinning cycle session, similar to an interval exercise, was performed in five patients with T1DM. The glucose measured via glucometer resulted in lower values when compared with the CGM system (4.1 ± 1.6 mmol·L^−1^ vs. 1.8 ± 0.7 mmol·L^−1^) during this exercise. No real conclusion was made due to the small sample size and lack of data to compare the accuracy.

It is commonly known that aerobic HIIE is similar, or even more superior, in enhancing oxidative capacity when compared to moderate CON in both healthy subjects [14,15] and in patients suffering from different chronic diseases [16,17,18]. These effects are suggested to be caused by markedly higher peak workloads during the intervals, resulting in an accumulation of mitochondrial proteins that drive mitochondrial biogenesis [18]. Growing interest in HIIE, as a model of exercise in healthcare, is seen due to the suggested greater improvements in health-related markers (e.g., cardiorespiratory fitness) in healthy subjects as well as those at risk for cardio-metabolic diseases. These welcomed benefits are simultaneously achieved with lower training volumes and training time [19]. Therefore, aerobic HIIE may be an alternative training method as opposed to the more standard CON. In patients with T1DM, HIIE may be associated with less of a decrease in glucose levels compared to CON [20]. The physiological mechanism responsible was suggested to be linked to counter-regulatory hormones, such as catecholamines and cortisol, promoting glycogenolysis. Furthermore, other metabolites (such as free fatty acids) were hypothesized to be associated with higher glucose levels during HIIE [21].

Considering the importance of CGM and the impressive effects of aerobic HIIE, studies investigating the accuracy of CGM during exercise, while matched for different mean intensities and durations, are entirely missing. The aims of this study were (1) to analyze the accuracy of CGM (Medtronic Enlite™, Guardian^®^ REAL-Time CGM, Medtronic Diabetes, Northridge, CA, USA) in comparison to capillary blood glucose measures during CON and HIIE at standardized mean exercise workloads [22]; and (2) to compare the influence of either CON or HIIE on the accuracy of CGM versus BG measures. We hypothesized that the difference between CGM and BG would be minimal and would be considered clinically acceptable. Additionally, it is hypothesized that the CGM system will perform measurements accurately no matter which type of exercise mode (CON vs. HIIE).

## 2. Materials and Methods

### 2.1. Subjects

Subjects were recruited by telephone calls, the contact information was provided from a database of volunteers with T1DM from the Medical University of Graz. The following general inclusion criteria were defined: male participants with the confirmed diagnosis of T1DM, diabetes duration over 12 months, aged between 18 and 35 years (inclusive), HbA1c < 8% (<64 mmol·mol^−1^), fasting c-peptide < 0.3 mmol·L^−1^, intensified insulin or insulin pump therapy, and no diabetic long-term complications. Testing day inclusion criteria were: no hypoglycemia 48 h pre-exercise and no alcohol consumption 24 h before testing. Exclusion criteria were defined as follows: illness or disease that could confound study results, use of drugs interfering with insulin action, addiction to alcohol, suspected allergy to trial products, mental incapacity, or other physical and/or mental diseases. Testing day exclusion criteria were: illness on or 24 h before the testing day, BG levels below 4.4 mmol·L^−1^ before testing, technical difficulties with the CGM system or incorrect time and/or amount of bolus insulin injection.

The study design was approved by the Ethics Committee of the Medical University of Graz with registry number 26-069 ex 13/14. The study protocol was published at ClinicalTrials.gov (ID: NCT02075567) [23] and performed according to Good Clinical Practice (GCP) and the Declaration of Helsinki. All subjects gave their written informed consent before participating in this trial.

### 2.2. Experimental Design

**Figure 1:**
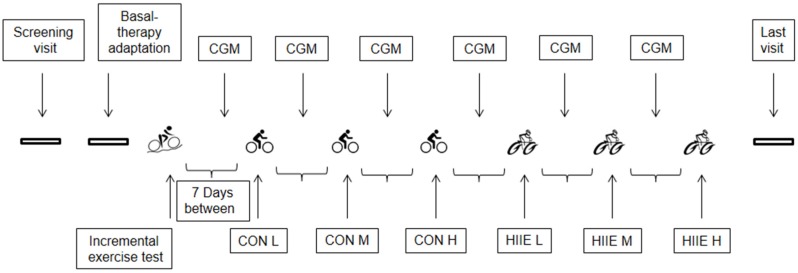
Timeline chart of the study. CGM = continuous glucose monitoring, CON = continuous exercise, HIIE = short high-intensity interval exercise. CON/HIIE L, M, H = low intensity, moderate intensity, high intensity CON/HIIE.

Overall, the study consisted of 24 visits for each subject (Figure 1). The first ten visits were used for the adaptation of basal insulin therapy [4]. At visit number 11, subjects had to perform a maximal incremental exercise test (IET) to determine the exercise intensities for the upcoming CON and HIIE. Visits 12, 14, 16, 18, 20, and 22 were used to assist subjects for the installation of the CGM system. Visits 13, 15, and 17 were used for performing CON, and visits 19, 21, and 23 were used for performing HIIE. Visit number 24 was the final visit. Data of seven subjects were available for the present analysis. One of the seven participants did not perform the HIIE at the highest mean exercise intensity due to time-dependent problems, and was excluded from statistical analyses for this mean exercise intensity. As an outcome variable we compared interstitial glucose levels measured by the CGM system to capillary blood glucose levels (reference values) during HIIE and CON.

#### 2.2.1. Adaptation of Long-Acting Insulin Therapy and Dietary Recording

To achieve homogeneity within the subjects, all patients were switched to basal insulin degludec (Tresiba, Novo Nordisk, Bagsvaerd, Denmark). Insulin degludec was injected once per day subcutaneously by the subject, with a starting dosage of 70% of their pre-study total daily basal insulin dose, modified according to Koehler et al. [24]. During a period of four weeks, an optimal setting for the basal insulin dose was attained. Additionally, dietary recordings were performed by the patients to obtain the usual carbohydrate intake. This individual amount of carbohydrates was used to match standardized meals prior to the exercise testing.

#### 2.2.2. Incremental Exercise Testing (IET)

At the start of the IET subjects sat on the cycle ergometer for 3 min without cycling (0 W) to control for day-to-day variability in cardiorespiratory and metabolic status. Afterwards they completed a 3 min active warm-up phase at 40 W before the workload was increased (quasi-ramp-protocol) by 20 W per minute until volitional exhaustion was achieved [25,26]. Immediately after exhaustion subjects continued by completing a 3 min active recovery period (40 W). In the end, subjects rested for an additional 3 min while passively sitting on the cycle ergometer (0 W). To prescribe individual exercise intensities for the CON and HIIE tests, maximal power (P_max_) and the first (LTP_1_) and second lactate turn point (LTP_2_) were determined. The LTP_1_ indicates the first increase in blood lactate concentration above resting values. The LTP_2_ is defined as the second abrupt lactate increase between LTP_1_ and P_max_. It is significantly higher than the LTP_1_ and related to the maximal lactate steady state [26]. LTP_1_ and the LTP_2_ were determined during the IET by means of a computer-based linear regression break point analysis. This was possible from the relationship of lactate to power output as the iteratively calculated intersection point between two regression lines, according to the three-phase model of energy supply [26]. P_max_ was determined at the point of voluntary exhaustion indicated by a drop of cadence below 40 rpm.

#### 2.2.3. Continuous Exercise (CON)

With one week in between, three CON tests with different standardized exercise intensities were conducted. Target workloads were set at the following exercise intensities: 5% of P_max_ from IET below the power at LTP_1_ (P_LTP1_) (L), 5% of P_max_ from IET above P_LTP1_ (M), and 5% of P_max_ from IET below the power at LTP_2_ (P_LTP2_) (H). These target workloads represent exercise intensities such as common low-intensity physical activity or occupational work for several hours (L), moderate walking or low-intensity running or cycling (M), and strenuous exercise near the maximal lactate steady state for a limited duration (H). Each test started with a 3 min resting period and a 3 min warm-up phase as in the IET. Exercise intensity was increased stepwise by 20 W·min^−1^ until the target workload (P_mean_) was reached and maintained for 30 min. Statistical analyses of CGM were only performed during that specific 30 min period. The following active and passive recovery periods of 3 min for each subject were the same as in the IET.

#### 2.2.4. High-Intensity Interval Exercise (HIIE)

**Figure 2:**
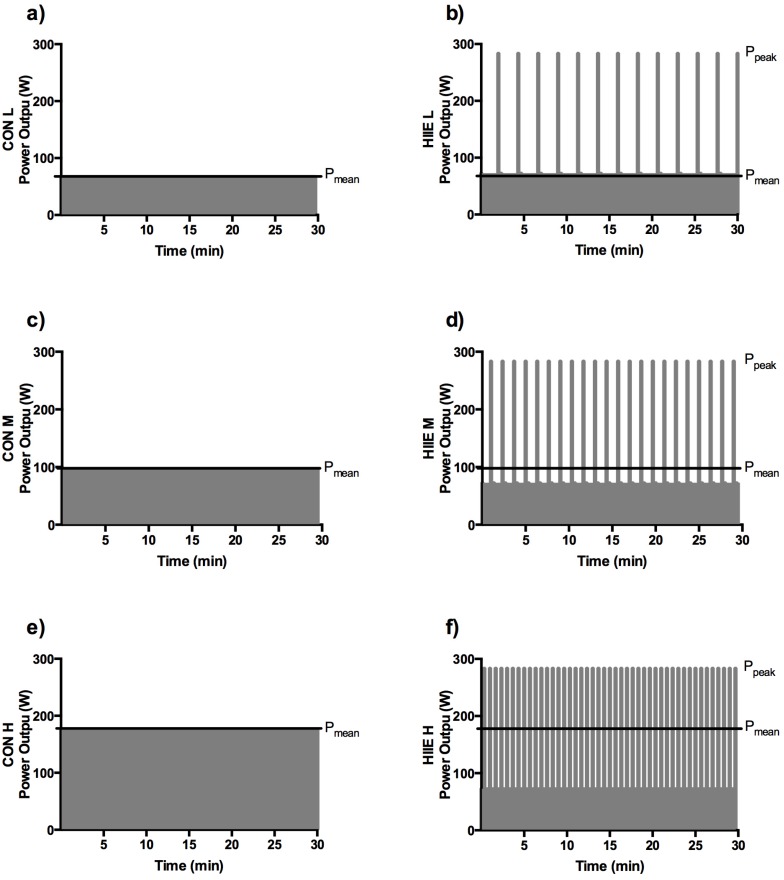
Prescription of the CON L (**a**), M (**c**), H (**e**); and HIIE L (**b**), M (**d**), H (**f**) exercise protocols matched for mean workload and exercise duration. All high-intensity intervals were the same in L, M, and H with P_peak_ set at P_max_ from the IET and t_peak_ was set at 20 s. P_mean_ was regulated by adapting t_rec_ from 1:6 (L) to 1:3 (M), and to 1:1 (H). P_mean_ = mean work load; P_peak_ = peak work load in the intervals; P_max_ = maximal workload from the incremental exercise tests (IET); t_peak_ = duration of peak exercise; t_rec_ = recovery time.

After the CON tests, three HIIE tests were performed with mean loads equal to the three target workloads of CON (L, M, H), also for a total duration of 30 min (Figure 2). The prescription of HIIE were based on the formula P_mean_ = (P_peak_ × t_peak_ + P_rec_ × t_rec_)/(t_peak_ + t_rec_) [22,25,27]. All HIIE tests were separated by one week. The initial procedure (resting period and warm-up phase) was identical to CON. For all HIIE tests, P_peak_ was set at P_max_ from the IET, and t_peak_ was set at 20 s. For L, recovery duration (t_rec_) was 120 s (work to rest ratio was 1:6); for M, t_rec_ was 60 s (work to rest ratio was 1:3); and for H, t_rec_ was 20 s (work to rest ratio was 1:1). The different P_mean_ workloads for L, M, and H in HIIE were managed by adapting t_rec_. The active recovery workload for L, M, and H was set just below P_LTP1_ (since P_LTP1_ was suggested to be the point of the optimal lactate clearance rate). Finally, the cool-down workload procedure was the same as for CON.

#### 2.2.5. Continuous Glucose Monitoring (CGM)

At the first CGM visit, subjects were passively equipped with a CGM system (Guardian^®^ REAL-Time CGM, Medtronic Diabetes, Northridge, CA, USA) to become familiar with its use. The sensor was inserted subcutaneously at least 24 h before exercise for a period of 48 h with a 2-h run-in calibration period. The CGM sensor (Medtronic Enlite™, Medtronic Diabetes, Northridge, CA, USA) was placed in the (lateral) abdominal tissue and measured interstitial glucose continuously [28], with a 5 min average being calculated from measurements every 10 s. Subjects were instructed to perform four calibrations within 24 h based on capillary glucose tests from the fingertip with their own glucometer. The last calibration was performed at least one hour before the exercise tests (CON and HIIE). The CGM system was additionally calibrated when required by the system.

During the CON and HIIE tests, interstitial glucose levels were read off the CGM system and immediately documented in the case report form (CRF) at the same time points as BG was taken from the earlobe (every 5 min). Furthermore, interstitial glucose values were downloaded after exercise. In case of a failure of the CGM during one of the tests (CON or HIIE), the data measured during the other exercise mode at the same mean exercise intensity were excluded (per protocol analysis).

#### 2.2.6. Carbohydrate Intake and Short-Acting Insulin Reductions

Each participant obtained a standardized liquid meal and an individualized short-acting insulin injection 4 h before the exercise tests. This time span was suggested to be adequate to avoid drastic glucose fluctuations close to the beginning of the exercise test, which might have influenced the accuracy of the CGM system during exercise. The calculated carbohydrate amount (59 ± 8 g) prior to the exercise tests were based on the average breakfast amount over the last four weeks before the study start. Standardized fluid clinical nutrition consisted of 39% carbohydrates, 36% proteins, and 25% fat of total grams (Fortimel Extra, Nutricia GmbH, Erlangen, Germany). Immediately after exercise, the same standardized meal ingestion and short-acting acting insulin injection as in pre-exercise conditions were administered. Short-acting insulin was reduced for CON and HIIE by 25% for exercise intensity L, 50% for M, and 75% for H, which is in accordance with Rabasa-Lhoret et al. [29].

### 2.3. Measurements

In all tests, capillary blood samples were taken from the ear lobe at rest, every 5 min during each specific exercise protocol, as well as at the end of each active and passive recovery periods to determine lactate and BG concentrations. These concentrations were determined by means of a fully enzymatic-amperometric method (Biosen S-line, EKF Diagnostics, Barleben, Germany), for the purpose of comparing to the interstitial glucose levels during the 30 min target workload period. Heart rate (PE 4000, Polar Electro, Kempele, Finland) and pulmonary gas exchange variables (ZAN 600, nSpire Health, Oberthulba, Germany) were collected continuously to control the target exercise intensity. Blood pressure measurements (every 2 min) and a 12-lead electrocardiogram were used in all tests for safety reasons. Hormone data are not shown since they have been published previously [4].

### 2.4. Statistical Analyses

Based on findings of a pilot-study, we made a sample size estimation for the correlation between both measurements (blood glucose and interstitial glucose during exercise) which gave a number of seven patients (correlation of *r* = 0.91 with a power of 0.95).

Distribution was tested via D’Agostino and Pearson omnibus normality tests. Descriptive statistics is given as mean ± standard deviation (SD). Comparison of means from CGM vs. BG was performed with paired *t*-tests, The Bland-Altman method (bias and 95% limits of agreement) and Pearson correlation. The CGM accuracy in CON vs. HIIE was compared with paired *t*-test of mean difference (MD) and mean absolute relative differences (MARD) of CGM and BG between exercise conditions per patient (CON L vs. HIIE L, CON M vs. HIIE M and CON H vs. HIIE H). Individual paired interstitial glucose and reference BG values are given using the Clarke Error Grid analysis [30]. Five zones characterize errors of varying levels of clinical significance, among which values in zones A and B are defined as “clinically acceptable”, whereas values in zones C, D, or E are considered potentially unsafe and could lead to clinically significant errors. Statistical analyses were performed with Microsoft Excel (Microsoft Corporation 2007, Washington, DC, USA) and standard software package Prism Software version 4.0 (GraphPad, La Jolla, CA, USA).

## 3. Results

### 3.1. Subjects’ Characteristics and Performance Data

Subjects were 24 ± 5.3 years old with the following anthropometric data: height 1.76 ± 0.40 m, weight 74 ± 5.1 kg, body mass index (BMI) 23.9 ± 2.5 kg·m^−2^. Diabetes-specific data were: diabetes duration 16.9 ± 8.1 years, HbA1c 7.4% ± 0.6% (57 ± 6.3 mmol·mol^−1^), c-peptide 0.13 ± 0.19 nmol·L^−1^, carbohydrate factor 12 ± 4.9 g [31], and total daily insulin dose 41 ± 16 U.

The IET revealed a maximal oxygen uptake (VO_2max_) of 52 ± 8.2 mL·kg^−1^·min^−1^ with an absolute P_max_ at 284.3 ± 43.1 W. P_LTP1_ was found at 82.1 ± 20.2 W and P_LTP2_ at 192.1 ± 33.6 W. Although P_mean_ (especially during CON H and HIIE H) was set at a very high mean exercise intensity, all subjects completed the pre-defined exercises. No hypoglycemia (BG < 3.9 mmol·L^−1^) occurred during any of the exercise sessions and no additional carbohydrate intake was needed.

### 3.2. Comparison of Accuracy in CGM and BG

**Figure 3:**
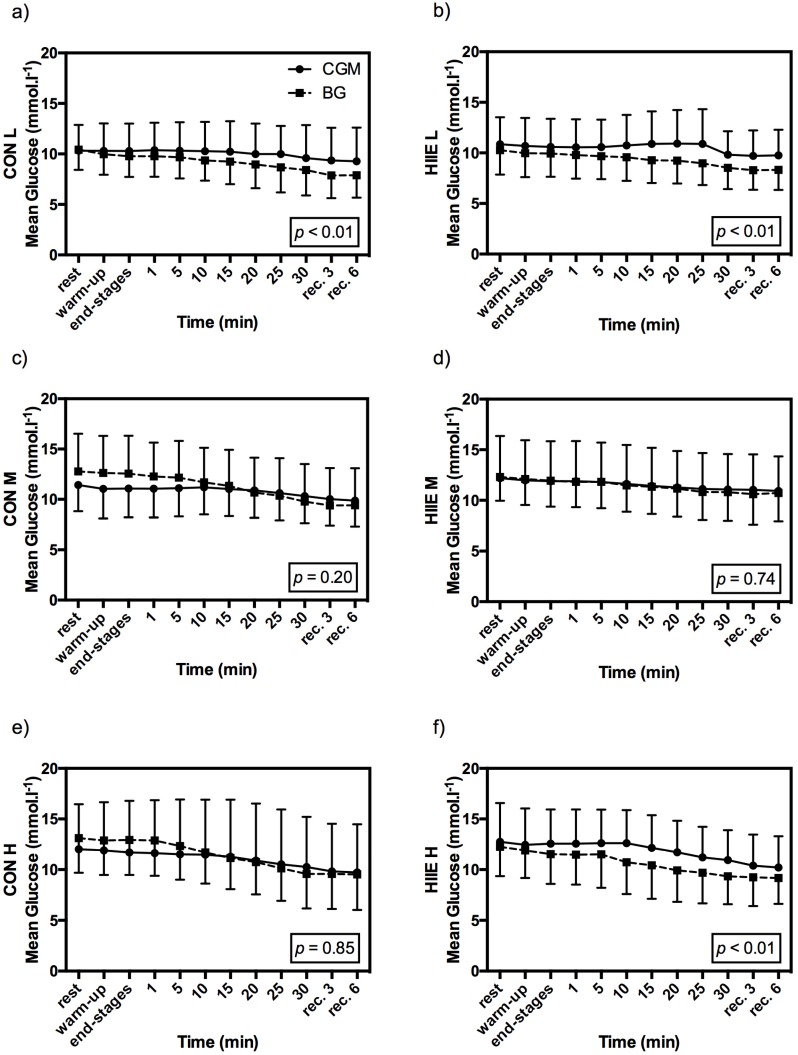
Glucose response from CGM (full line) and BG (dotted line) during CON and HIIE for the three standardized mean exercise intensities given as mean ± SD: CON for exercise intensities L (**a**), M (**c**), and H (**e**); as well as HIIE for exercise intensities L (**b**), M (**d**), and H (**f**). *p*-values are given for the comparison of mean BG and mean CGM.

All data were normally distributed. Of 504 expected comparable pairs of CGM and BG values, 489 values (97%) were available between CON and HIIE. In 15 of the pairs (3%) the sensor signaled a failure. CGM overestimated mean glucose values during CON for exercise intensity L (*p* < 0.01) and during HIIE for exercise intensities L (*p* < 0.01) and H (*p* < 0.01). No significant differences were found for mean glucose values during CON in M (*p* = 0.20) and in H (*p* = 0.85), as well as for HIIE in M (*p* = 0.74) (Figure 3).

During all exercise tests, significant correlations were found between CGM and BG. Dependence for CON exercises were: *r* = 0.93; *p* < 0.01 (L), *r* = 0.92; *p* < 0.01 (M), and *r* = 0.96; *p* < 0.01 (H). Similar significant responses in correlations of CGM and BG were found in HIIE: *r* = 0.74; *p* < 0.01 (A), *r* = 0.99; *p* < 0.01 (B), and *r* = 0.91; *p* < 0.01 (C).

**Figure 4:**
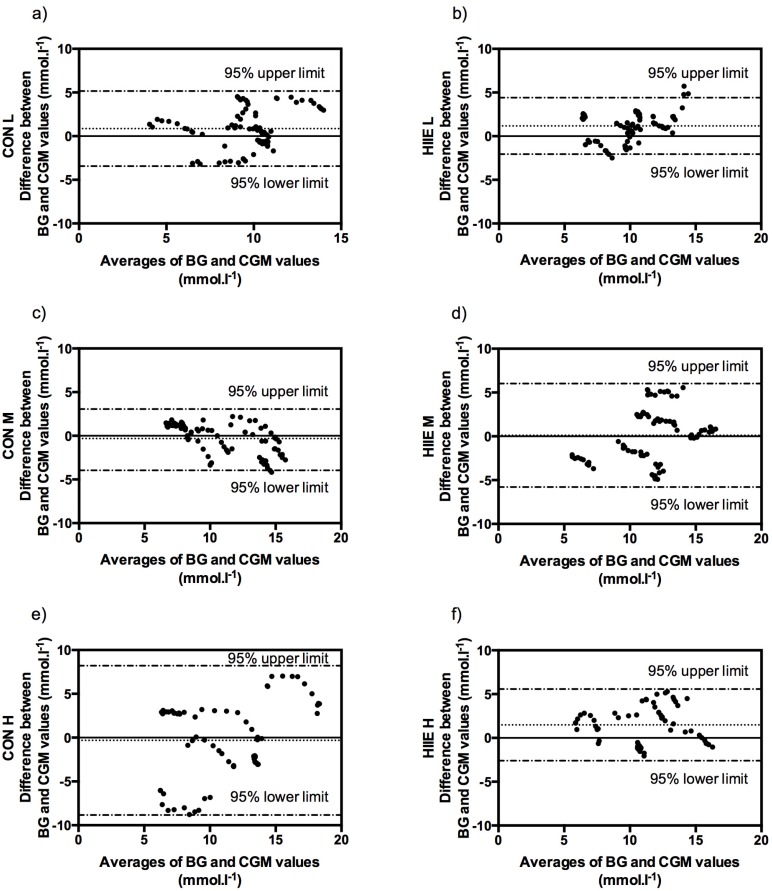
Bland-Altman plots for the comparison of BG and CGM values during CON and HIIE for the three standardized mean exercise intensities given as bias (dotted line) and levels of agreement (dot-dashed line): CON for exercise intensities L (**a**), M (**c**), and H (**e**); as well as HIIE for exercise intensities L (**b**), M (**d**), and H (**f**).

The Bland-Altman method derived bias and levels of agreement for absolute values of glucose at exercise intensities L, M, and H were found at (CGM to BG) 0.85 (−3.44, 5.15) mmol·L^−1^, −0.45 (−3.95, 3.05) mmol·L^−1^, and −0.31 (−8.83, 8.20) mmol·L^−1^ in CON and at 1.17 (−2.06, 4.40) mmol·L^−1^, 0.11 (−5.79, 6.01) mmol·L^−1^, and 1.48 (−2.60, 5.57) mmol·L^−1^ in HIIE (Figure 4).

### 3.3. Influence of Exercise Type on CGM

**Figure 5:**
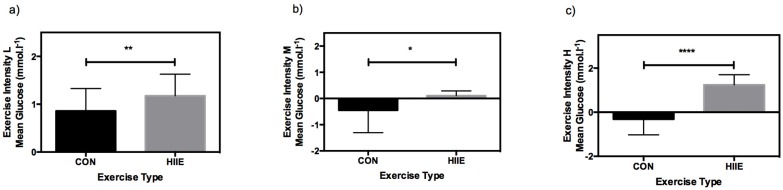
Mean differences in glucose response (CGM vs. BG) in comparison of CON (black bars) and HIIE (grey bars) given as means ± SD. Levels of significance for the different exercise intensities were: L (**a**) (** *p* = 0.001), M (**b**) (* *p* = 0.01), and H (**c**) (**** *p* < 0.0001).

With respect to the MD of CGM and BG, significant differences were found between CON and HIIE for all mean exercise intensities (Figure 5). In comparison of CON versus HIIE, MARD was 19.8% ± 14.5% vs. 16.9% ± 9.1%, *p* = 0.13 (L), 12.8% ± 8.2% vs. 26.5% ± 17.6%, *p* < 0.0001 (M), and 23.7% ± 10.8% vs. 15.5% ± 10.8%, *p* = 0.001 (H).

A trend in overestimation of CGM in HIIE can be clearly detected. No such trend was found in CON, as CGM did overestimate glucose levels for L, but not for M or H.

### 3.4. Clinical Acceptance of CGM in Comparison to BG

**Figure 6:**
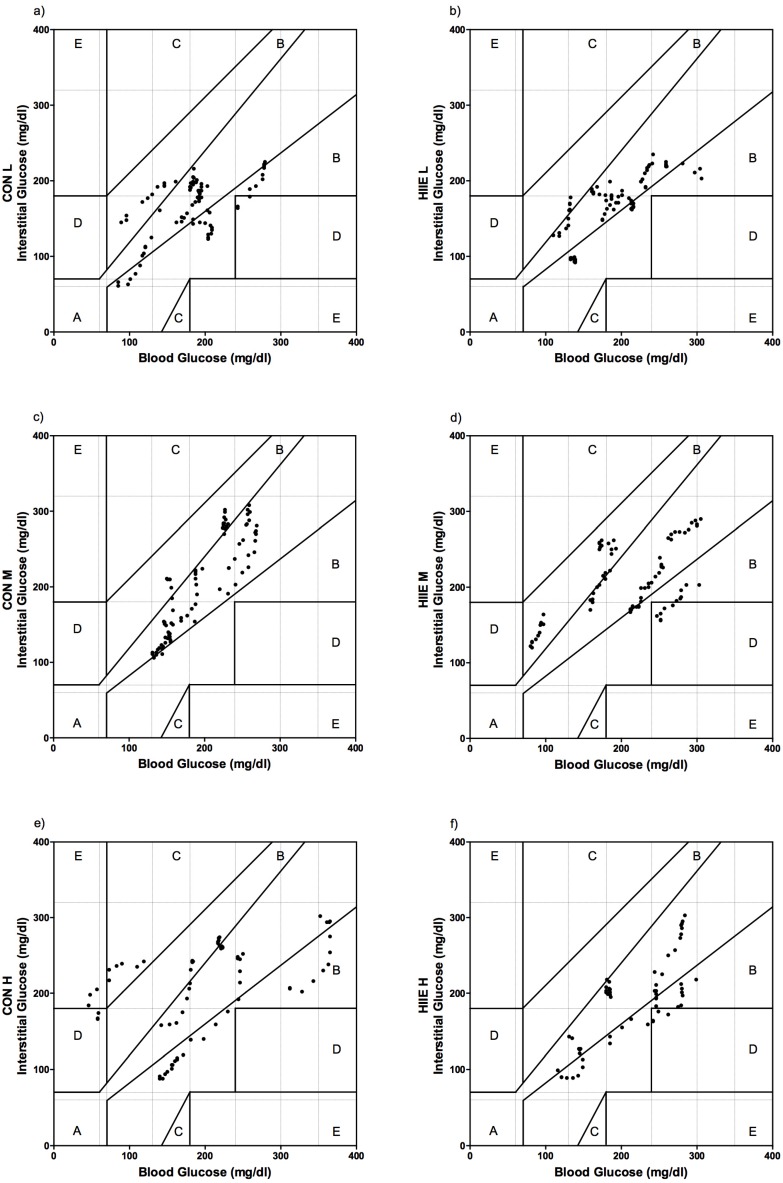
The Clarke Error Grid analyses given for the three standardized exercise intensities. CON for exercise intensities L (**a**), M (**c**), and H (**e**); as well as HIIE for exercise intensities L (**b**), M (**d**), and H (**f**).

The Clarke Error Grid analysis showed that during CON, 96% of values were in zones A and B (52% and 44%) for mean intensity L, 100% (79% and 21%) for M, and 78% (30% and 48%) for H. During HIIE, zones A and B were reached for exercise intensity L in 100% (63% and 37%), for M in 93% (48% and 45%), and for H in 93% (55% and 38%) (Figure 6).

## 4. Discussion

Although all data were significantly correlated, significant differences were found between some of the exercises when comparing CGM and BG (determined by means of a paired *t*-test). These findings are in agreement with those of Iscoe et al. [13]. The MD and MARD of the comparison of CGM accuracy in CON and HIIE also showed significant differences. The differences found in half of all tests (CON L, HIIE L, and HIIE H) might be explained by the overall physiological lag of ~3–12 min due to the diffusion of glucose across the capillary endothelial barrier and the glucose rate-limiting membrane [32]. It is critical that this delay be considered for practical implications, even more so when exercising with low glucose levels.

In comparison to the type of exercise, CGM tended to overestimate glucose levels during HIIE, which is contrary to the findings of Bally et al. [11], the only other study investigating the accuracy of CGM during CON and HIIE. Comparing our results to this study is difficult, since they prescribed exercise intensities as percentages of VO_2max_, which may have led to inhomogeneous cardiorespiratory and metabolic responses of their subjects [26,33]. The Bland-Altman method displayed minor differences with no definitive information of a systemic inaccuracy of CGM. Even so, we found wide limits of agreement, especially for CON H. This finding is in line with the results of Herrington et al. [8], but contrary to the findings of Fayolle et al. [12].

Clinically speaking, the most important result was found in the Clarke Error Grid analysis, showing no clinically-relevant inaccuracy between measures of CON and HIIE. Within CON H, 78% of the data was detected in zones A and B, which must be considered clinically unacceptable, due to poor accuracy. Since exercising leads to an increased skin blood flow due to metabolic heat production, heat will be transferred to the peripheral regions [34]. This transfer mechanism could serve as an explanation for the accurate balance between BG and interstitial fluid in most of our data [9]. In our study, exercise intensity was defined as percentages of submaximal markers (% of LTP_1_ and LTP_2_) [26] and P_mean_ and exercise duration were matched between both exercise types [4], which enabled a comparison of the CGM accuracy in CON vs. HIIE. In comparison to healthy subjects, Herrington et al. [8] concluded that CGM should only be used in conjunction with a standard glucometer since CGM systems do not meet the accuracy standards from International Organization for Standardization (ISO). Although our data can be interpreted to support sufficient accuracy of a CGM system, in some of the exercise sessions, we also recommend using CGM only concomitantly with the standard glucometer measures for safety reasons.

Regarding CGM accuracy during exercise our data showed clinically-acceptable accuracy for different mean CON and HIIE intensities (except for CON H close to LTP_2_), which is further confirmed by significant correlations for all tests. Additionally, statistically significant differences were found between the means of CGM and BG concentrations, as well as displaying a wide levels of agreement with the Bland-Altman method. Therefore, one must be critical of CGM for practical exercise training use.

To the best of our knowledge no study investigated the accuracy of CGM at the maximal lactate steady state, just below the LTP_2_ in patients with T1DM. Workloads, as used in our study, are increasingly becoming greater focuses of interest, since patients with T1DM are not restricted to low-intensity exercise training, and are also competing like healthy athletes [35,36]. Ninety percent of endurance athletes with T1DM mainly check their glucose levels in pre- and post-exercise conditions, but only 50% reported to check glucose levels during exercise [37]. As we could show recently [4], BG rapidly decreases after the onset of exercise. During this phase, it is of the highest importance to accurately track changes in glucose concentration in order to adapt the therapy or pacing adequately. CGM systems offer this optimal opportunity to measure glucose continuously during exercise.

During intense exercise, insulin has to be reduced [4] and/or carbohydrates have to be supplemented [35] to avoid hypoglycemia due to the activation of glucose-transporter type 4 (GLUT 4) caused by insulin injection and muscle contraction. For our study, it is important to note that elevated glucose concentrations did not influence the accuracy of the CGM during exercise. We may, therefore, recommend using CGM in addition to a standard glucometer in physically active patients with T1DM, especially during exercise, to ensure euglycemia.

The effects of exercise on HbA1c still remain controversial in patients with T1DM. There is no clear evidence that long-term glucose levels were reduced due to exercise [1]. Exaggerated and long-lasting reduction of insulin dose, that might result from the fear of hypoglycemia [38], leads to high pre-exercise glucose levels, which accounts for the absence of the reduction in HbA1c levels. Under standard care use, CGM is associated with an improvement in metabolic control (indicated by a lower HbA1c) [39]. Taking that into account, CGM may additionally enhance the positive effects of exercise on long-term glucose levels.

Calibrating the CGM system by means of the participants’ own measurement systems had no influence on the results during exercise. This was validated by comparison to the Biosen S-line system data (EKF Diagnostics, Barleben, Germany). Participants’ own measurement systems data for resting conditions were not significantly different (*p* = 0.967), and significantly correlated to the Biosen S-line system data (EKF Diagnostics, Germany) (*r* = 0.96; *p* < 0.0001).

A limitation to our study was the use of only one type of CGM sensor (Medtronic Enlite™, Medtronic Diabetes, Northridge, CA, USA). Therefore, the transfer of our findings to other existing CGM devices is not possible. An additional limitation of our study is the fact that we did not randomize the order of the different exercise tests. Habituation to the type of exercise may reduce the stress response-decreasing hormones, such as catecholamines and cortisol. These stress hormones increase hepatic glucose production and decrease peripheral glucose uptake [1,40], therefore, the accuracy of the CGM system might have been influenced due to the chosen order of our tests. On the other hand, the subjects were used to regulate activity which was evident by their catecholamine and cortisol response at rest, as well as their heart rate response during the warm up (which remained the same for all tests), and did not significantly change throughout the study [4]. Our results cannot be transferred to resting conditions, as we analyzed CGM accuracy only during the exercise sessions. Additionally it has to be considered that the earlobe is not a usual site to perform capillary BG tests; although, it was already shown that differences in earlobe and fingertip glucose did not demonstrate any clinical significant difference [41].

Furthermore, it was shown recently that acetaminophen might falsely elevate CGM values compared to BG values. The use of acetaminophen was not prohibited in our study but subjects were asked for medications routinely. None of the subjects indicated any additional medication (including acetaminophen) during the 48 h pre- and 24 h post-exercise period [42]. However, these specific side effects should be considered in upcoming research investigating CGM accuracy. Further research should investigate the CGM accuracy under a wider range of glucose concentrations, because our standardized protocol largely minimized the usual glucose variations (including hypoglycemic events). Upcoming studies should also investigate the accuracy of different types of CGM systems during CON and HIIE at standardized exercise intensities prescribed in relation to submaximal markers (e.g., LTP_1_ and LTP_2_). Furthermore, the effects of exercise interventions on long-term glycemic response, inflammation and diabetic comorbidities (with and without the use of CGM) are still not proven in detail.

## 5. Conclusions

In conclusion, this is the first study investigating the effects of CON and HIIE on the accuracy of CGM and BG with exercise intensities prescribed as percentages of submaximal markers (LTP_1_ and LTP_2_), and with P_mean_- and duration-matched exercise modes. Clarke Error Grid analysis provided that the applied CGM system (Guardian^®^ REAL-Time CGM, Medtronic Diabetes, Northridge, CA, USA) using a Medtronic Enlite™ Sensor (Medtronic Diabetes, Northridge, CA, USA) is clinically acceptable, except for CON H. Even so, limits of agreement from the Bland-Altman method and paired *t*-test showed differences for CGM with BG during CON and HIIE. The CGM system may be suggested as an additional supportive tool along with glucometer measurements, but should be applied with care to avoid glycemic disturbances during exercise in patients with T1DM. Further, larger studies are needed for determining standard use recommendations in order to assure safe exercise practices among those with T1DM.

## Abbreviations

The following abbreviations are used in this manuscript:

T1DM	Type 1 diabetes mellitus
BG	Blood glucose
CGM	Continuous glucose monitoring
P_mean_	Mean workload power
CON	Continuous exercise
HIIE	High intensity interval exercise
HbA1c	Glycated hemoglobin (A1c)
C-peptide	Connecting peptide
GCP	Good Clinical Practice
P_max_	Maximal power output
LTP_1_	First lactate turn point
LTP_2_	Second lactate turn point
P_LTP1_	Power output at the first lactate turn point
P_LTP2_	Power output at the second lactate turn point
L	Mean exercise intensity 5% of P_max_ from IET below P_LTP1_
M	Mean exercise intensity 5% of P_max_ from IET above P_LTP1_
H	Mean exercise 5% of P_max_ from IET below P_LTP2_
P_peak_	Peak workload during HIIE
t_peak_	Peak workload duration during HIIE
P_rec_	Recovery load during HIIE
t_rec_	Recovery duration during HIIE
CRF	Case report form
SD	Standard deviation
MD	Mean difference
MARD	Mean absolute relative difference
BMI	Body mass index
VO_2max_	Maximal oxygen uptake
W	Watt
GLUT 4	Glucose-transporter type 4
ISO	International Organization for Standardization
